# *Oroxylum Indicum* ameliorates chemotherapy induced cognitive impairment

**DOI:** 10.1371/journal.pone.0252522

**Published:** 2021-06-03

**Authors:** Satyanarayana R. Pondugula, Mohammed Majrashi, Mohammed Almaghrabi, Sindhu Ramesh, Kodye L. Abbott, Manoj Govindarajulu, Kristina Gill, Eddie Fahoury, Natasha Narayanan, Darshini Desai, Jun Ren, Rishi Nadar, Trey McElroy, Timothy Moore, Muhammed Majeed, Nagabhushanam Kalyanam, Muralikrishnan Dhanasekaran

**Affiliations:** 1 Department of Anatomy, Physiology and Pharmacology, College of Veterinary Medicine, Auburn University, Auburn, Alabama, United States of America; 2 Department of Drug Discovery and Development, Harrison School of Pharmacy, Auburn University, Auburn, Alabama, United States of America; 3 Department of Pharmacology, Faculty of Medicine, University of Jeddah, Jeddah, Saudi Arabia; 4 School of Pharmacy, University of Wyoming College of Health Sciences, Laramie, Wyoming, United States of America; 5 Sabinsa Corporation, East Windsor, New Jersey, United States of America; King Abdulaziz University, SAUDI ARABIA

## Abstract

While chemotherapy is the most effective therapeutic approach for treating a variety of cancer patients, commonly used chemotherapeutic agents, often induce several adverse effects. Escalating evidence indicates that chemotherapeutics, particularly doxorubicin (DOX) and cyclophosphamide (CPS), induce cognitive impairment associated with central nervous system toxicity. This study was performed to determine neuroprotective effects of *Oroxylum indicum* extract (OIE) in regard to preventing chemotherapy induced cognitive impairment (CICI) occurring after 4 cycles of DOX (2mg/kg) and CPS (50mg/kg) combination chemotherapy in male C57BL/6J mice. OIE significantly prevented the chemotherapy impaired short-term cognitive performance, exploratory behavior associated with cognitive performance, cognitive performance, and spatial learning and memory in the Y-maze, Open-Field, Novel Object Recognition, and Morris Water Maze tests, respectively. These data suggest that OIE protects from the CICI. OIE decreased the reactive oxygen species and lipid peroxide generated by the chemotherapy treatment in the brain, while also blocking the chemotherapy-induced glutathione depletion. These results establish that OIE exhibits potent antioxidant activity in chemotherapy treated mice. Notably, OIE significantly increased the Complex-I and Complex-IV activities in the brain, indicating that OIE enhances mitochondrial function in the brain. In silico analysis of the major active chemical constituents (Oroxylin A, Baicalein and Chrysin) of OIE indicated that OIE has a favorable absorption, distribution, metabolism and excretion (ADME) profile. Taken together, our results are consistent with the conclusion that OIE prevents CICI by counteracting oxidative stress and perhaps by improving mitochondrial function.

## Introduction

As per the American Cancer Society estimates, 1.8 million Americans will be diagnosed with cancer in 2020, in addition to 14.5 million cancer survivors. Trends in five year survival rate of almost all cancer patients have increased significantly [1]. This increase in survival can be attributed to a combination of improvements in both diagnosis and treatment. Among the available treatment options, chemotherapy is the most effective therapy for treating a variety of cancer patients [2]. Unfortunately, chemotherapy often results in undesirable cognitive side effects for patients, resulting in decrease in dose or termination of chemotherapy. One example of a cognitive side effect is “mental fog,” which is measurable as difficulties with learning, memory, and executive functioning [3–5]. This chemotherapeutics induced cognitive impairment (CICI) is also known as “chemobrain,” frequently develops during chemotherapy and persists following termination of treatment, thus severely affecting the quality of life. [4, 6]. This chemotherapy induced neurotoxicity limits the dosing regimen, decreasing its effectiveness, or even cessation of treatment [4]. Presently, no evidence-based therapy or preventative interference for CICI exists [3]. It is imperative that efforts are made to develop novel therapeutic strategies to prevent the occurrence of CICI. Epidemiologic studies have validated that the consumption of botanicals significantly reduces the overall risk for cancer [7]. It would prove highly beneficial if a novel botanical could be discovered to protect from CICI.

*Oroxylum indicum* extract (OIE) is a traditional herbal medicine belonging to the family Bignoniaceae and is predominantly grown in tropical countries such as in India, China, and Japan [8]. It constitutes flavonoids namely Oroxylin A, Baicalein and Chrysin. OIE possesses a broad spectrum of pharmacological activities, hence various parts of the plant have been used in the treatment of multiple disorders such as cancer, fever, hepatitis, and diarrhea [9]. OIE has been utilized as antidiuretic, antispasmodic, aphrodisiac, anti-diarrheal, antipyretic, antitussive and preventing other respiratory illnesses. Currently, there are no studies that have established the neuroprotective effects of OIE against CICI. The molecular mechanism of CICI is believed to be due to several factors, including chemotherapy induced oxidative stress, mitochondrial dysfunction, and activation of microglia (caused by pro-inflammatory cytokines) [10].

Consequently, the antioxidant properties and mitochondrial activity enhancing potential form a valid scientific basis to investigate the neuroprotective properties of OIE to diminish CICI [11]. Therefore, we set to determine the neuroprotective effects as well as the antioxidant and mitochondrial function enhancing mechanisms of OIE that may allow OIE to overcome the chemotherapeutic-induced neurotoxic events. Computational analysis was also performed to determine the absorption, distribution, metabolism and excretion (ADME) profile of the flavonoids of OIE, indicating the improved bioavailability of OIE.

## Materials and methods

### Chemicals

The following chemicals/drugs were purchased from Sigma Chemical Company (St. Louis, MO., USA): bovine serum albumin (BSA), NADH, Cytochrome C, kynuramine, DCF dye, ethylenediaminetetraacetic acid (EDTA) sodium salt, O-phthaldialdehyde (OPT), Tris-buffered saline tablet, Phosphate buffered saline tablet, Reduced glutathione (GSH), dimethyl sulfoxide (DMSO), doxorubicin (DOX), cyclophosphamide (CPS), and hydrogen peroxide (H_2_O_2_).

### Animals

Male C57BL/6J mice were purchased from The Jackson Laboratory. All *in vivo* experiments were implemented following approval from Auburn University’s Institutional Animal Care and Use Committee (IACUC). Eight weeks old male C57BL/6J mice were accommodated in a temperature-controlled room with a 12-h day and night cycle with free access to food and water. Food intake and body weight were measured twice a week during experimental phase in all experiments. A total of 10 mice per group (total of 50 mice) were used in the study. The mice (25–35 g) were administered with vehicle saline (group I); chemotherapeutics (CT), doxorubicin (2 mg/kg) and cyclophosphamide (50 mg/kg) intraperitoneally with one injection per week for a total of four weeks (group II); *Oroxylum Indicum* extract (OIE, Sabroxy^®^) prepared by Sabinsa Corporation [250 mg/kg OIE low dose (LD) (group III) and 500 mg/kg OIE high dose (HD) (group IV)] was mixed with powdered rodent food and fed daily for 4 weeks to mice treated with CT. The Oroxylum indicum bark extract used in this investigation was provided by Sabinsa Corporation using the methods described in US patent number: US10555982B2. The identified major bioactives in this extract included Oroxylin A, Baicalein, and Chrysin. The OIE treatment alone group received 500mg/kg of OIE in powdered rodent food (group V). The control mice also received the powdered rodent food for 4 weeks without OIE.

### General behavioral changes

The general behavioral changes were monitored regularly. The behavioral studies were performed 2 weeks following treatment completion. Animals were monitored by two trained behavioral observers using a well-established protocol [12].

### Behavioral markers associated with cognitive impairment

#### Open field test

In this study, the time spent in the center was monitored in the Open field apparatus as per our previously published protocol [13].

#### Y-maze test

In this study, the percentage entry into the novel arm was monitored as per our previously published protocol [13].

#### Novel Object Recognition (NOR) test

In this study, the novel object recognition was monitored as per our previously published protocol [13].

#### Morris Water-Maze (MWM) test

Animals were subjected to visible training platform for the first 3 days by setting up a pool measuring 1.2 m in diameter and 50 cm deep. The optimal temperature of water was maintained to 24 ± 2 °C. The width of the platform was 104 cm, which was similar for both hidden and visible trials. There were three visible platform trials performed on day 1 and day 3. Animals unable to escape the platform within 3 min, were manually directed to the platform. Probe session and training phase (day 2 and 4) were performed to determine the time spent in the quadrant where the platform was previously present. Animals were returned to their respective cage (3 trials) after using the heating pad to prevent hypothermia. Animals were delivered to either of the quadrants, with the target quadrant labeled as north, randomly. Visual cues were placed above the pool in each quadrant [14].

### Biochemical assays

#### Tissue preparation

Mice from each group were sacrificed by decapitation in the morning to avoid diurnal variations of endogenous amines, enzymes, and other antioxidant molecules. The brain was dissected out, flash frozen in liquid nitrogen and stored at −80 °C. Cortex and rest of the brain homogenates were used for biochemical studies. The tissue lysates were prepared by homogenizing in phosphate buffer saline (0.1M, pH 7.8), using a glass-Teflon homogenizer, followed by centrifugation at 10,000g for 60 min at 4 °C and the supernatant was collected as previously described [13].

#### Protein estimation

Protein was estimated using the Thermo Scientific Pierce 660 nm Protein Assay reagent kit (Pierce, Rockford, IL) [15].

#### Determination of ROS generation

Reactive oxygen species generation was estimated spectrofluorometerically by our previously published protocol [13]. The generation of ROS was measured, normalized to tissue total protein content, and reported as relative fluorescence intensity/mg protein.

#### Estimation of lipid peroxidation content

Colorimetric procedure using thiobarbituric acid was utilized to measure thiobarbituric acid reactive substance content using the plate reader (BioTek Synergy HT plate reader). Lipid peroxide reacts with thiobarbituric acid to form a chromophoric thiobarbituric acid reactive substance (TBARS) that can be measured spectrophotometrically at 532nm. A standard curve was obtained using MDA and the findings were expressed as uM/mg protein [13].

#### Glutathione (GSH) content estimation

Fluorimetric procedure was utilized to measure glutathione content using the plate reader (BioTek Synergy HT plate reader, BioTek, VT, USA) at 340nm (excitation wavelength) and 420nm (emission wavelength). Glutathione content measurement in the control and drugs treated samples was based on the condensation reaction between OPT and glutathione, where the product formed were measured fluorimetrically. A standard curve was obtained using glutathione and the findings were expressed as glutathione uM /mg protein [13].

#### Determination of mitochondrial Complex-I activity

NADH oxidation to NAD+ is catalyzed by mitochondrial Complex-I (NADH dehydrogenase). The conversion of NADH to NAD+ was measured spectrophotometrically at 340 nm [16].

#### Determination of mitochondrial Complex-IV activity

Cytochrome C oxidation is catalyzed by mitochondrial Complex-IV (Cytochrome C oxidase). Cytochrome C oxidation was spectrophotometrically measured in the homogenized tissues of all the groups at 550 nm [16].

#### Computational analysis

Drug molecules with favorable absorption, distribution, metabolism, and elimination (ADME) properties are the primary indicators of successful candidate molecules in drug discovery and development. In this study, QikProp filter from Schrödinger was used to calculate several pharmacokinetic and pharmacodynamic properties of Oroxylin A, Baicalein and Chrysin. The Qikprop set of descriptors (SASA, FOSA, FISA, PISA, #metabolites, CNS distribution, QPlog BB, Donor HB, Accept HB, logP, % human oral absorption, and Rule of 5) were selected to describe this aspect of the compounds permeability, metabolism and activity. Schrödinger Release 2019–2: QikProp, Schrödinger, LLC, New York, NY was used in the computational analysis.

### Statistical analysis

Data are presented as means ± SEM. Statistical analyses and data were assessed using one-way analysis of variance (ANOVA) followed by Dunnet’s multiple comparisons test (P < 0.05 was considered to be statistically significant). Statistical analysis was performed using Prism-V software (La Jolla, CA, USA).

## Results

The control as well as chemotherapy (CT), *Oroxylum indicum* extract (OIE), chemotherapy + low dose of OIE (CT+OIE-LD), and chemotherapy + high dose of OIE (CT +OIE-HD) treated mice groups were monitored regularly for several behavioral parameters (Table 1). Animals were monitored by two trained behavioral observers using a well-established protocol [10]. There was no significant change in body weight among the treatment groups. OIE consumption did not result in any adverse events, major toxicity, or death. Furthermore, OIE also had no significant adverse effects pertaining to the Central Nervous System (CNS), gastrointestinal system (GIT) and genitourinary system. Similar to OIE, CT treatment did not result in noticeable adverse events.

**Table 1:** 

Behavioral Parameters	Control	OIE (500mg/kg)	Chemotherapeutics (CT)	CT + OIE LD	CT + OIE HD
Abnormal posture (head press)	N	N	N	N	N
Aggressive behavior (fight)	N	N	N	N	N
Agitation	N	N	N	N	N
Allergic reaction (redness of the skin/ eye)	N	N	N	N	N
Anaphylactic shock/death	N	N	N	N	N
Body weight (% increase in 5 weeks)	17%	6.7%	5%	5.8%	5.2%
Bowel movement	N	N	N	N	N
Diarrhea	None	None	None	None	None
Drooling	N	N	N	N	N
Emesis	None	None	None	None	None
Eye bulging	N	N	N	N	N
Fighting (Aggressive behavior)	N	N	N	N	N
Grinding teeth/Chattering	N	N	N	N	N
Grooming	Y	Y	Y	Y	Y
Hair coat erection	N	N	N	N	N
Head twitching	N	N	N	N	N
Hematuria	N	N	N	N	N
Hind limb abduction	N	N	N	N	N
Hyperactivity (excessive jumping)	N	N	N	N	N
Licking body	N	N	N	N	N
Licking genitals	N	N	N	N	N
Mortality observed	N	N	N	N	N
Locomotion (increase/decrease)	N	N	N	N	N
Narcolepsy	N	N	N	N	N
Open mouth breathing	N	N	N	N	N
Penile erection (stimulatory behavior)	N	N	N	N	N
Rapid Breathing	N	N	N	N	N
Salivation / Drool	N	N	N	N	N
Seizure	N	N	N	N	N
Sniffing	N	N	N	N	N
Stool color	N	N	N	N	N
Straub tail	N	N	N	N	N
Sunken eyes/lack of blinking	N	N	N	N	N
Tremor	N	N	N	N	N
Tumor	N	N	N	N	N
Wiggling Whiskers	N	N	N	N	N

### Effect of CT &/or OIE on exploratory behavior

**Fig 1:**
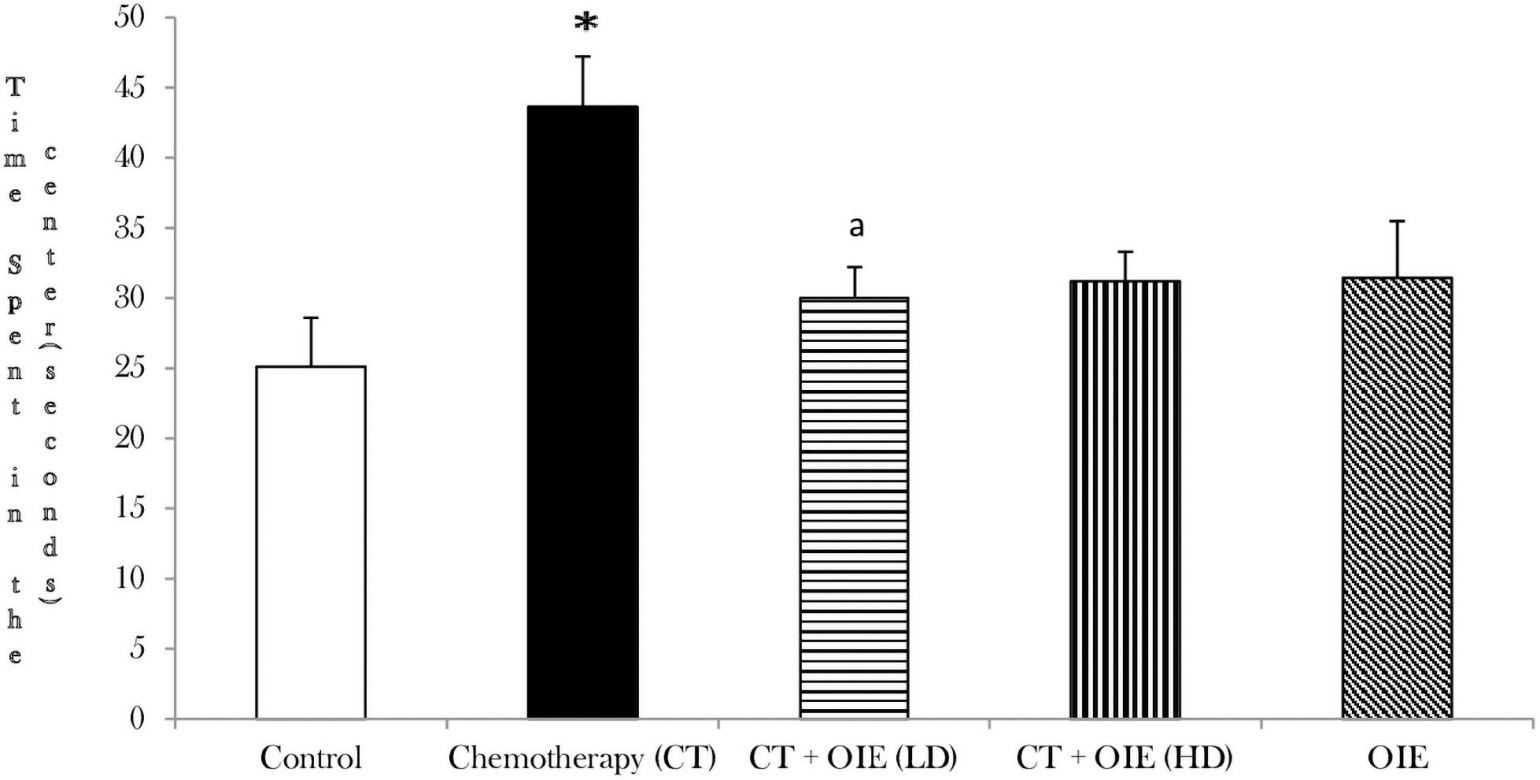
Open field test: Effects of CT &/or OIE on exploratory behavior. Time spent in the center was recorded in the open field test. Results shown are expressed as the mean ± SEM (n = 10) *p<0.05 vs. control mice, ^a^*p*<0.05 vs. CT mice.

To investigate the effects of OIE on exploratory behavior, the open-field test was carried out. CT treatment significantly increased the amount of time spent in the center as compared to the control in the open field (n = 10, *p<0.05). However, feeding with OIE low dose (LD) significantly reduced the amount of time spent in the center of the open field by CT+OIE-LD mice as compared to the CT treated mice (n = 10, ^a^*p*<0.05, Fig 1). Although we saw a reduction in the amount of time spent in the center of the open field in CT+OIE-HD treated mice in comparison to CT treated mice, it was not statistically significant (*p*>0.05). These findings suggest that OIE-LD significantly improves the exploratory behavior impaired by CT treatment.

### Effect of CT &/or OIE on short-term cognitive performance in the Y-maze test

**Fig 2:**
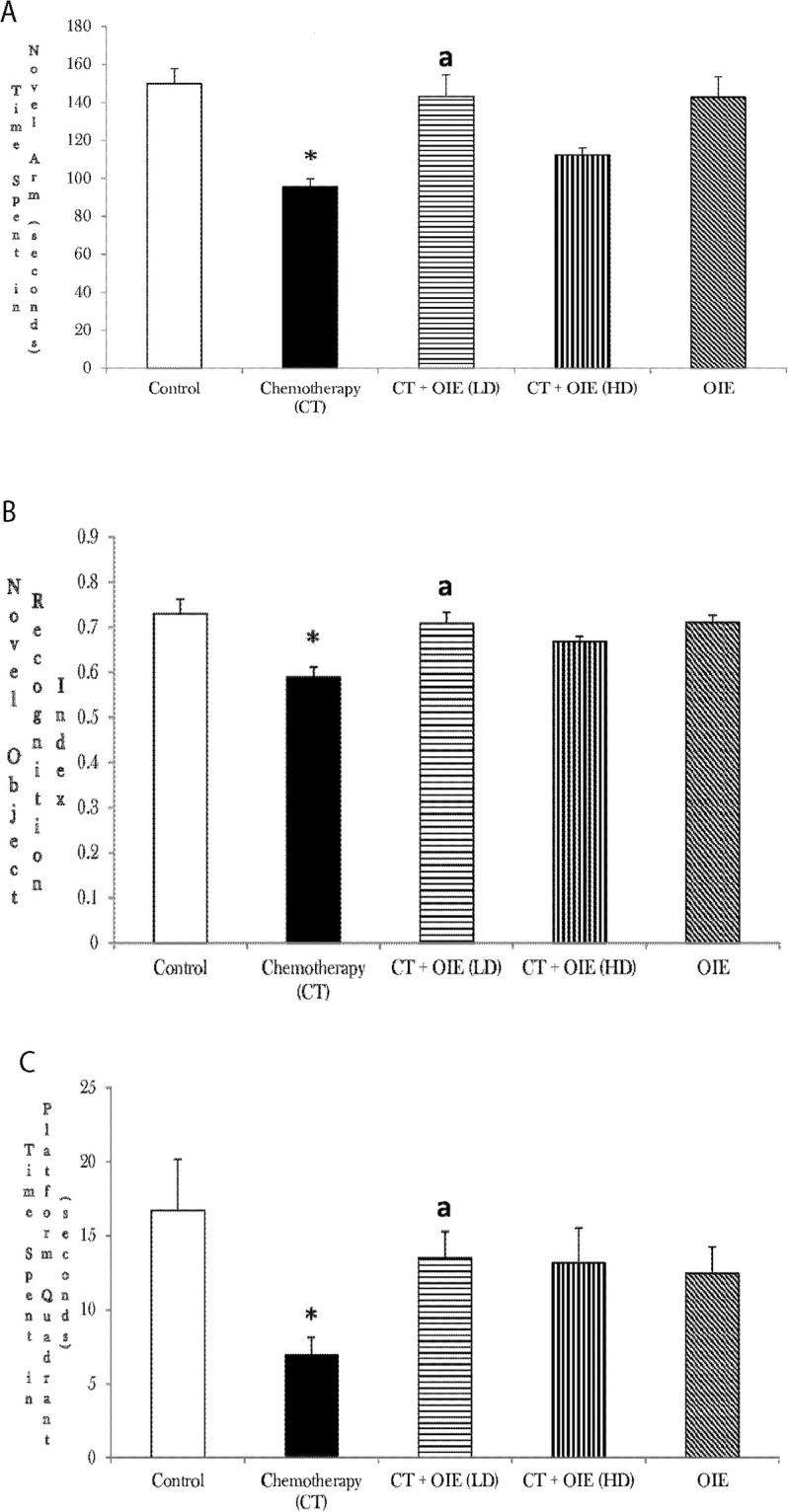
Effect of CT &/or OIE on spatial and recognition memory. (a) Y-maze test to determine spatial memory indicating time spent in the novel arm. (b) Recognition memory determined by NOR. Novel Object Recognition Index is a ratio of number of touches on the novel object / total number of touches on the familiar and novel object. (c) Morris water maze test for spatial learning and memory. Time spent in the platform quadrant was recorded. Results shown are expressed as the mean ± SEM (n = 10) *p<0.05 vs. control mice, ^a^*p*<0.05 vs. CT mice.

To elucidate the cognitive deficits improved by treatment with OIE in CT treated mice, we performed hippocampal-dependent short-term memory in Y-maze test. CT treated mice spent a significantly decreased time in the novel arm of the Y-maze as compared to the control mice (n = 10, *p<0.05). However, feeding with low dose of OIE (OIE-LD) significantly increased the time spent in the novel arm in the CT treated mice (n = 10, ^a^*p*<0.05, as compared to the CT only treated mice, Fig 2a). Similar to open field, CT+OIE-HD treated mice did not show statistically significant improvement in time spent in the novel arm in comparison to CT treated mice (*p*>0.05). These results indicate OIE LD significantly improves short-term memory impairment in CT treated mice.

### Effect of CT &/or OIE on cognitive performance in the NOR test

This test was done to examine the effects of OIE on object recognition memory. Novel Object Recognition Index is a ratio of Number of Touches on the Novel Object / Total Number of Touches on the familiar and novel Object. The effect on NOR was confirmed by comparing the discrimination index, which revealed a significant improvement in the CT+OIE-LD mice compared to CT mice in overall performance in NOR (n = 10, ^a^*p*<0.05, as compared to the CT, Fig 2b). Though we saw an improvement in CT+OIE-HD mice in overall performance in NOR compared to CT only mice, it was not statistically significant (*p*<0.05).

### Effect of CT &/or OIE on spatial learning and memory in the MWM test

To examine the effects of OIE on spatial learning and memory we used the MWM. CT treatment significantly decreased the time spent in platform Quadrant as compared to the control (n = 10, *p<0.05). CT+OIE-LD significantly increased the time spent in the platform Quadrant in the CT treated mice (n = 10, ^a^*p*<0.05, as compared to the CT, Fig 2c). Similar effects were seen with CT+OIE-HD group as compared to CT only group; however, the results were not statistically significant (*p*>0.05). This data suggests that OIE LD protects from CT impaired spatial learning and memory.

### Effect of CT &/or OIE on the levels/activities of oxidative stress markers (prooxidants and antioxidants)

The effects of OIE on the levels of pro-oxidative markers such as ROS, lipid peroxide as well as effects of OIE on antioxidant levels (glutathione) were examined in the cerebral cortex and rest of the brain.

**Fig 3:**
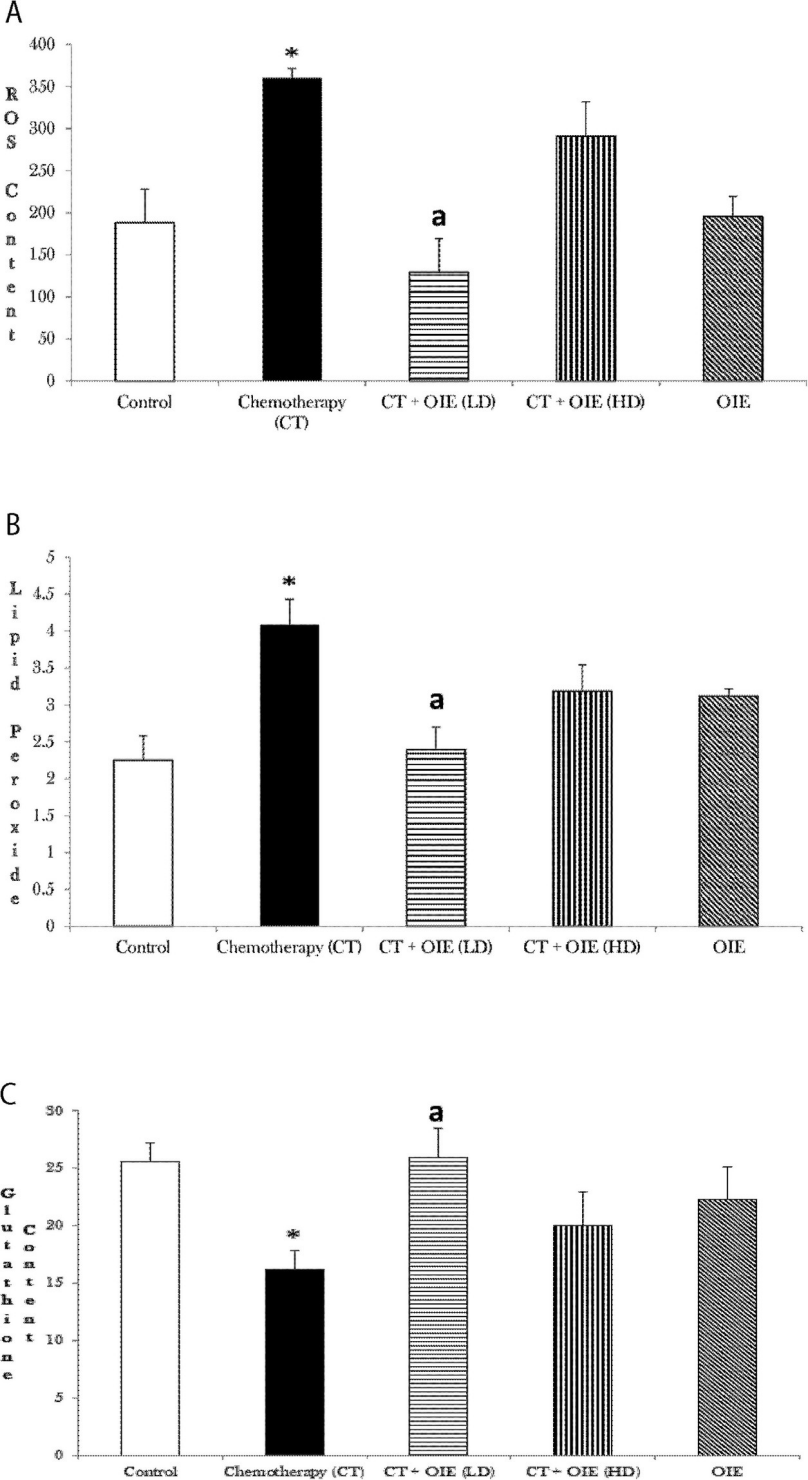
Effect of CT &/or OIE on the levels of ROS, lipid peroxidation and Glutathione content in the cortex. (a) The levels of oxidative stress marker-ROS in the cortex were measured spectrofluorimetrically as relative fluorescence units (492/527 nm)/mg protein. (b) Lipid peroxide formation was measured as TBARS formed (532 nm)/mg protein. (c) Glutathione content was measured spectrofluorimetrically and expressed as GSH content (μM)/mg protein, mean ± SEM (n = 5) *p<0.05 vs. control mice, ^a^*p*<0.05 vs. CT mice.

CT treatment significantly increased the generation of ROS and lipid peroxidation, whereas it significantly depleted the glutathione content in the cerebral cortex (n = 5, *p<0.05, Fig 3a–3c). Treatment with OIE-LD significantly decreased the ROS (Fig 3a, ^a^*p*<0.05) and lipid peroxide (Fig 3b, ^a^*p*<0.05) generated by CT in the cerebral cortex. Similar effects were noted with treatment with OIE-HD; however, the results were not statistically significant. Furthermore, OIE-LD significantly blocked the CT-induced glutathione depletion in the cortex (Fig 3c, ^a^*p*<0.05). CT+OIE-HD treated mice showed increase in glutathione content, however the results were not statistically significant (p>0.05).

**Fig 4:**
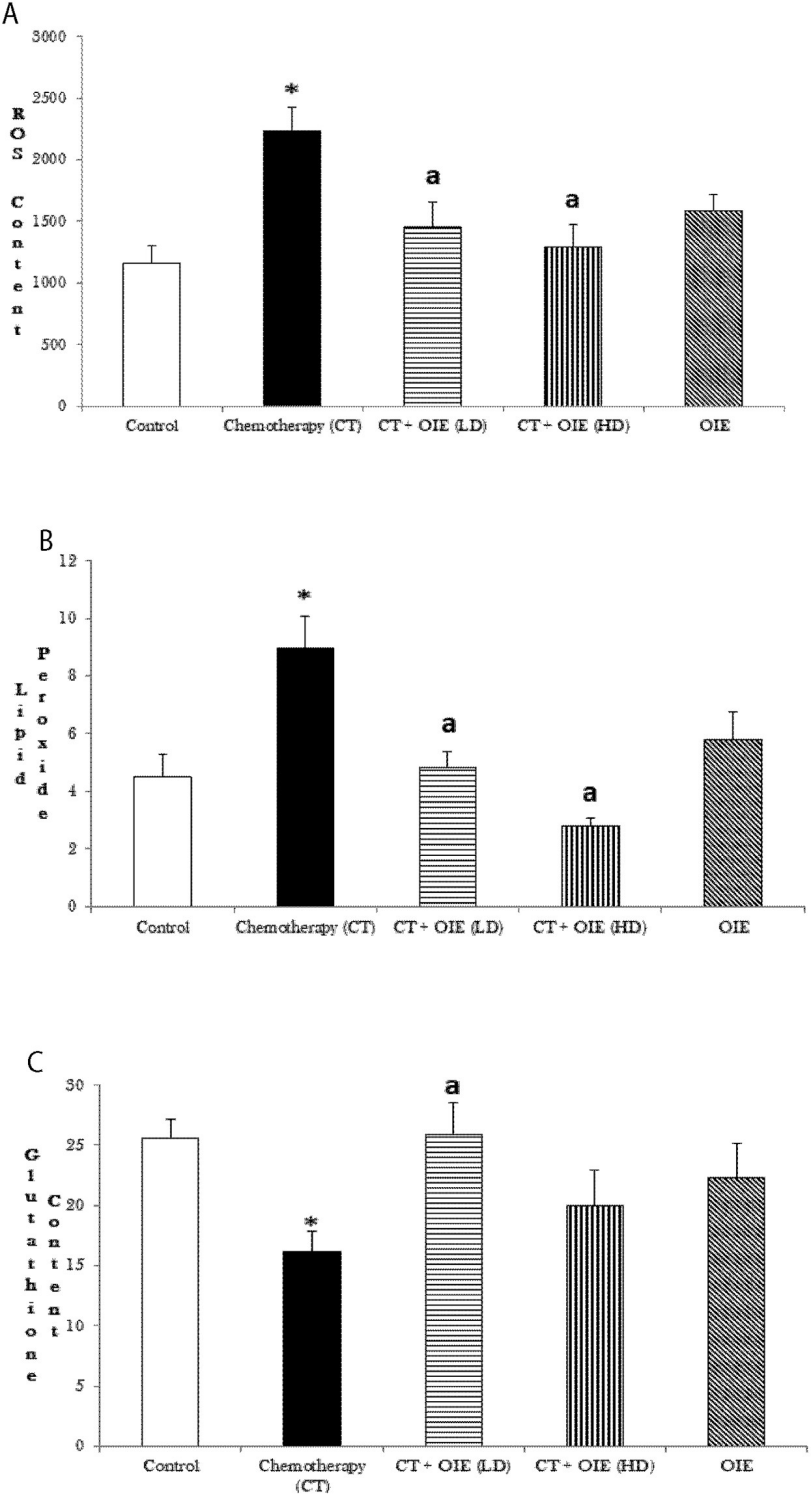
Effect of CT &/or OIE on the levels of ROS, lipid peroxidation and Glutathione content in the rest of brain. (a) The levels of oxidative stress marker-ROS in the cortex were measured spectrofluorimetrically as relative fluorescence units (492/527 nm)/mg protein. (b) Lipid peroxide formation was measured as TBARS formed (532 nm)/mg protein. (c) Glutathione content was measured spectrofluorimetrically and expressed as GSH content (μM)/mg protein, mean ± SEM (n = 5) *p<0.05 vs. control mice, ^a^*p*<0.05 vs. CT mice.

With respect to the rest of the brain, CT treatment significantly increased the generation of ROS and lipid peroxidation, whereas it significantly depleted the glutathione content (n = 5, *p<0.05, Fig 4a–4c). Treatment with OIE-LD and OIE-HD significantly decreased the ROS (Fig 4a, ^a^*p*<0.05) and lipid peroxide (Fig 4b, ^a^*p*<0.05) generated by CT in the rest of the brain. Furthermore, OIE-LD significantly improved the CT-induced glutathione content depletion in the rest of the brain (Fig 4c, ^a^*p*<0.05). We did not see a statistically significant improvement in glutathione content in the rest of the brain of CT+OIE-HD treated mice in comparison to CT treated only mice (p>0.05).

### Effect of OIE on the mitochondrial function (Complex-I & Complex-IV activities)

**Fig 5:**
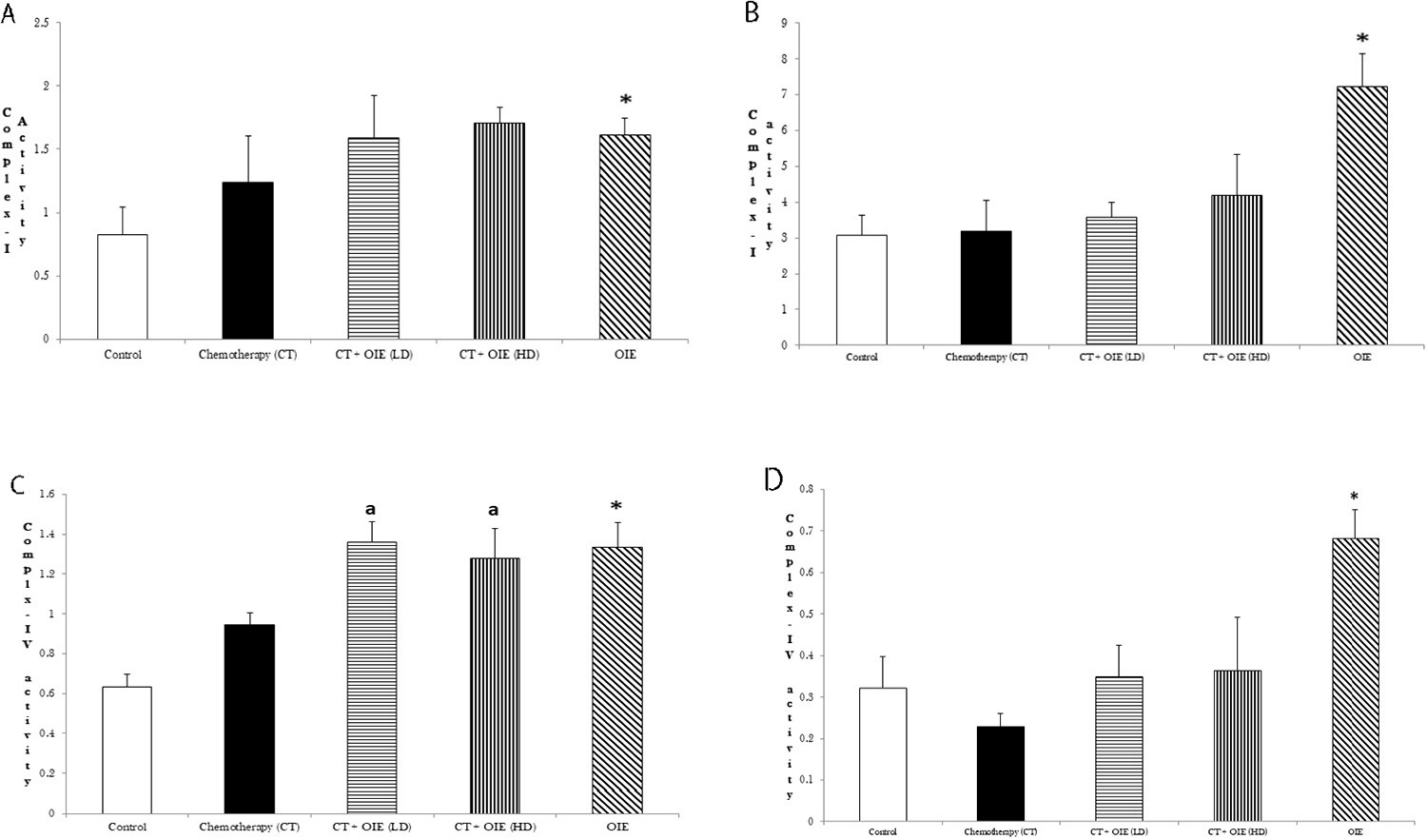
Effect of OIE on the mitochondrial function-Complex-I and Complex-IV activity. Complex-I activity was measured spectrophotometrically using NADH as substrate in the cortex (a) and the rest of the brain (b). Results are expressed as NADH oxidized (μM)/mg protein. Complex-IV activity was measured spectrophotometrically using cytochrome-C as substrate in the cortex (c) and the rest of the brain (d). Results are expressed as cytochrome-C oxidized (μM)/mg protein, mean ± SEM, (n = 5) *p<0.05 vs. control mice, ^a^*p*<0.05 vs. CT mice.

The effects of OIE on the Complex-I and Complex-IV activities were determined in the cerebral cortex and rest of the brain. OIE alone significantly increased the Complex-I activity in the cortex and rest of the brain tissue as compared to the control (n = 5, *p<0.05, Fig 5a and 5b). Similarly, OIE significantly increased the Complex-IV activity in the cortex and rest of the brain tissue as compared to the control (Fig 5c, 5d). Together, these observations suggest that, OIE enhances mitochondrial functions in the cortex and rest of the brain.

### *In-silico* analysis of chemical constituents of OIE

Solvent accessible surface area (SASA) of a molecule is its surface area that is in contact with the solvent in the biological system. SASA refers to that molecule is in contact to a biomolecule like a protein or a membrane, hence there is a possibility for better absorption and bioavailability. Other parameters like hydrophobic components of the SASA (FOSA), hydrophilic components of the SASA (FISA), and π (carbon and attached hydrogen) components of the SASA (PISA) values for Oroxylin A were all within the acceptable range as displayed in Table 2. All other parameters which include CNS, QPlogBB, % human oral absorption and Lipinski’s rule of five were within the range of drug bioavailability (Tables 2 and 3) are favorable parameters for the administration of OIE to patients.

**Table 2:** 

Compound	SASA	FOSA	FISA	PISA	#metab	CNS	QPlogBB
Oroxylin A	502.213	83.645	127.834	290.734	3	-1	-0.746
Baicalein	483.691	0	185.948	297.743	3	-2	-1.263
Chrysin	473.628	0	147.680	325.948	2	-1	-0.8

The permissible ranges are as follows: Area are (300–1000), FOSA: Hydrophobic components of the SASA (0.0–750.0), FISA: Hydrophilic components of the SASA (7.0–330.0), PISA: π (carbon and attached hydrogen) components of the SASA (0.0–450.0), #metab: Number of likely metabolic reactions (1–8) CNS: −2 (inactive) to +2 (active), QPlog BB: (−3.0 to −1.2) polar compounds have large negative values. SASA: Solvent Accessible Surface.

**Table 3:** Calculated ADME properties of the molecules.

Compound	mol MW	Donor HB	Accept HB	log P	% Human Oral Absorption	Rule of Five
Oroxylin A	284.268	1	3.75	2.548	91.689	0
Baicalein	270.241	2	3.75	1.694	76.823	0
Chrysin	254.242	1	3	2.3	87	0

The permissible ranges are as follows: Mol weight: (130–725), Donor HB: (0.0–6.0), Accept HB: (2.0–20.0), LogP: (−2.0 to 6.5), % Human oral absorption: >80% high, <25% low, Rule of five (maximum 4).

## Discussion

Based on the existing literature, the current study is the first to illustrate the novel neuroprotective effects of OIE against CICI in *in vivo* and *in-silico* analysis showing favorable ADME of Oroxylin A, Baicalein and Chrysin. The neuroprotective studies of OIE were established using several valid behavioral and biochemical studies. For the behavioral studies, open field test, Y-maze, NOR, MWM were used to validate the cognitive protective effects of OIE. The open field test is an accepted qualitative and quantitative scientific behavioral measure of exploratory performance and general activity in rodents [17]. Intriguingly, it measures several facets of rodent behavior beyond simple locomotion. Primarily, the open field is an enclosure of different shapes (commonly square, rectangular, or circular) with surrounding confines that prevent the escape of rodents. The general fundamental and common outcomes of open field are the movement related rodent behavior such as distance moved, time spent in the center or in the edges, rearing, and change in general activity over a period. The most common outcomes measured are the time spent in center and locomotor activity (hyper/hypo) and defecation likely gauge some aspects of stress and anxiety. However, Y-maze, NOR and MWM are applied to evaluate the short-term memory in rodents [18, 19]. Spontaneous alternation in the arms of the Y-maze is a measure of spatial working memory. Rodents commonly explore the arms of the maze but are propelled by an innate curiosity to explore previously unvisited arms. Like Y-maze, MWM is a cognitive behavioral experiment for evaluation of spatial learning behavior in rodents that relies on distal cues to navigate from sites around the perimeter of an open swimming arena to trace a submerged escape platform. Rodents with an intact prefrontal cortical / hippocampal function have a suitable spatial working memory and spends more time in the unvisited arms or traces the submerged escape platform quickly. Furthermore, in addition to cognitive functions, these behavioral tests are regularly used as tools to assess the sedative, toxic, or stimulant effects of synthetic drugs and botanical/herbal products. Thus, the behavioral studies carried out are valid tools to establish the cognitive protective effects of OIE. CICI significantly induced cognitive behavioral deficits. OIE significantly reduced the CICI in mice, as assessed by the Open field, Y-maze, NOR and MWM tests (Figs 1 and 2a–2c).

In our study, CICI, like other toxins such as metals (mercury, lead, aluminum, zinc, cadmium overload), pesticides / petrochemicals (polychlorinated biphenyls, polychlorinated dibenzo-p-dioxins, brominated flame retardants, Bisphenol A and phthalates), assorted synthetic chemical agents (nicotine, alcohol, mycotoxins), persistent air pollutants (nitrogen dioxide-NO_2_, black carbon) and therapeutic drugs (anticholinergics, antidepressants, anticonvulsants, histamine H_2_ antagonists, corticosteroids, nonsteroidal anti-inflammatory drugs, cardiac medications) significantly retarded the cognitive function [20–23]. Oxidation is critical for maintaining homeostasis of metabolic process to perform normal physiological process. However, when there is abnormal ROS accumulated, it can create oxidative stress leading to pathophysiological changes as in neurodegenerative diseases and cancers by attacking biologically related structures. Trang et al., 2018 have demonstrated the potent antioxidant activity of OIE [24]. The naturally occurring flavonoids are known to possess cognitive enhancing and neuroprotective properties [25–27]. Baicalin being an important flavonoid component, possesses potent neuroprotective and cognitive enhancement effects by varying molecular mechanisms [28] such as attenuation of the elevation of excitatory neurotransmitters (glutamate and aspartate), attenuation of apoptosis, as well as decreasing the areas of infarction in cerebral ischemic models [29, 30]. In another study performed by Mairuae et al., OIE protected human neuronal cells against oxidative stress, cellular injury and β-amyloid injury by attenuating the oxidative stress (generation of ROS, increasing superoxide dismutase and catalase activity), and suppressing apoptosis (caspase activity), increasing the phosphorylation of Akt and cAMP-responsive element binding protein, and resulting in the increase in expression of Bcl-2 protein [31]. Mechanistically, we show that chemotherapy increased oxidative stress and induced cognitive deficits (Figs 1–3). Notably, OIE reduced the chemotherapy-induced increase in ROS and lipid peroxide levels in the cortex and rest of the brain (Figs 3 and 4). It has been shown that doxorubicin and cyclophosphamide induced neurotoxicity is associated with oxidative stress [32]. Mitochondrial Complex-I and Complex-IV are indices of mitochondrial health and is a measure of the function of the cell to produce energy [33–36]. Notably, there are evidences that mitochondrial dysfunction, as noted by decreased Complex I and IV, is associated with cognitive impairment in Alzheimer disease [37]. Inciting our hypothesis that mitochondrial dysfunction is one of the key mechanisms of neurodegeneration and cognitive deficits, we show here that treatment with OIE significantly increased the mitochondrial functions (Fig 4) in addition to improving the behavioral deficits (Figs 1 and 2), suggesting an underlying relation between mitochondrial protection and neuroprotection. High mitochondrial density present in neurons are vulnerable to oxidative stress [38]. A decrease in mitochondrial function has also been associated with cognitive impairment in neurodegenerative diseases [39] and dementia [40, 41]. Therefore, we suggest that the OIE can increase synaptosomal mitochondrial integrity, possibly by enhancing the Complex-I and Complex-IV activities leading to ATP production [42] and thereby increasing the availability of mitochondrial energy under demanding conditions. Consequently, this may result in increased pro-oxidants against oxidative damage in neuronal connections involved in cognition and memory. Based on our current data, we propose that antioxidants and mitochondrial protectants may represent a striking, efficacious treatment for chemotherapy-induced neurotoxicities. It would be beneficial if a botanical could be shown to harbor neuroprotective properties capable of reducing CICI [43, 44]. The neuroprotective effects of OIE are mainly attributed to its antioxidant effect due to decrease in the pro-oxidant and increase of the antioxidant which led to decreased lipid peroxidation and furthermore OIE also significantly increased the function of the mitochondria. Therefore, OIE can be used to reduce chemotherapeutic induced cognitive impairment. The current investigation was one of the first studies to evaluate the amelioration of cognitive impairment induced by chemotherapeutics, by restoration of tissue redox balance and improving the mitochondrial functions. These results are in harmony with previous studies of neuroprotection against the oxidative stress induced by chemotherapeutics [45–47]. A study performed by Ren et al. determined the pharmacokinetics of Oroxylin-A [48]. We also have further established with the computational analysis, that OIE (mainly contains oroxylin-A, baicalein, and chrysin) is a safe and effective product with appropriate absorption, distribution, metabolism, and elimination profile for human use.

Taken together, our results indicate that systemic administration of a prooxidant and mitochondrial protectant such as OIE may be capable of decreasing the CICI. Hence, we propose that the prevention of oxidative stress and mitochondrial toxicities due to chemotherapy is an important therapeutic target to prevent cancer treatment-induced neurotoxicities. The addition of OIE to combination chemotherapy may well represent a promising novel therapeutic approach to prevent the progress of neurotoxicities in patients treated for cancer.
